# Evaluation of the potential impact and cost-effectiveness of respiratory syncytial virus (RSV) prevention strategies for infants in Argentina

**DOI:** 10.1016/j.vaccine.2024.126234

**Published:** 2024-10-03

**Authors:** Gonzalo Guiñazú, Julia Dvorkin, Sarwat Mahmud, Ranju Baral, Clint Pecenka, Romina Libster, Andrew Clark, Mauricio T. Caballero

**Affiliations:** a Centro INFANT de Medicina Traslacional (CIMET), Escuela de Bio y Nanotecnología (EByN), Universidad Nacional de San Martín (UNSAM), Buenos Aires, Argentina; b Fundación INFANT, Buenos Aires, Argentina; c Consejo Nacional de Investigaciones Científicas y Técnicas (CONICET), Argentina; d Department of Health Services Research and Policy, Faculty of Public Health and Policy, London School of Hygiene and Tropical Medicine, London, UK; e Center for Vaccine Innovation and Access, PATH, Seattle, Washington, USA.

**Keywords:** Cost-effectiveness, Immunization, Low-income and middle-income countries respiratory syncytial virus, Decision model

## Abstract

**Background.** New interventions are available for the prevention of respiratory syncytial virus (RSV) disease in young infants. We aimed to assess the potential impact and cost-effectiveness of using a long-acting monoclonal antibody (RSV mAb) or maternal RSV vaccine in the Argentine context. **Methods.** We used a static proportionate outcomes model to calculate the costs and consequences of using RSV mAb or maternal RSV vaccine over a ten-year period (2025–2034) in Argentina, assuming both year-round and seasonal administration. We compared each intervention to no pharmaceutical RSV intervention. The primary outcome was the discounted cost per disability-adjusted life year (DALY) averted from a societal perspective. We assumed willingness-to-pay of US$ 12,285 per DALY averted (0.9 times the national gross domestic product per capita). We used population study data on costs and disease burden and the efficacy of clinical trials of both interventions as inputs. We ran deterministic and probabilistic uncertainty analyses. **Findings.** Either strategy (RSV mAb or maternal RSV vaccine) could prevent >25% of RSV deaths aged <5 years and ∼30% aged <6 months (the age group where most intervention impact occurs). With a dose price of $US 50, both products have a 100% probability of being cost-effective compared to no intervention (US$ 5283 [95%CI $5203–$5363] and US$ 5522 [95%CI $5427 – $5617] per DALY averted for year-round use of RSV mAb and maternal RSV vaccine, respectively). Similar health impact could be achieved by a six-month seasonal strategy, which could improve cost-effectiveness by around 45% (assuming the dose price is unchanged). **Interpretation.** Either RSV mAb or maternal RSV vaccine are worth consideration in Argentina when priced at ≤US$ 50 per dose. A seasonal strategy could improve cost-effectiveness.

## 1 Introduction

Respiratory Syncytial Virus (RSV) is a common respiratory pathogen that can affect people of all ages [1]. The burden of RSV disease is particularly pronounced among the youngest and poorest children in low- and middle-income countries (LMICs) [[2], [3], [4]]. Over 90% of RSV-related fatalities take place in LMICs, with nearly 70% of these deaths occurring outside health facilities [2]. Argentina, an upper-middle income country according to the World Bank has an infant mortality rate of 8.4 deaths per 1000 live births and maternal mortality of 44.9 deaths per 100,000 live births [5]. In Argentina, RSV was confirmed in 16% of all deaths in children 1–23 months of age [6]. The majority of fatal RSV cases did not receive prompt medical care and died at home [4].

RSV places a substantial financial burden on healthcare systems, families, and economies worldwide [[7], [8], [9]]. The annual global economic burden of RSV is estimated to be approximately €4·82 billion among children under the age of five [7]. Notably, a significant portion of this burden (65%) is borne by LMICs, underscoring the disproportionate impact on regions with fewer resources [7]. In Argentina, each infant admitted to a public hospital with RSV acute lower respiratory tract infection (ALRTI) costs approximately US$587, resulting in Government expenditure exceeding US$13 million per year [9].

Currently, three approved preventive interventions offer protection against RSV in young infants. Palivizumab, a humanized IgG1 monoclonal antibody (mAb), exhibited efficacy against RSV only in high-risk premature infants after the administration of five doses with a 30-day interdose interval. The high price-per-dose (PPD) of Palvizumab means it is not feasible to use in most LMICs [10]. Two alternative interventions have recently demonstrated efficacy against RSV-related ALRTI [[11], [12], [13]]. Nirsevimab (Beyfortus™, Astra Zeneca and Sanofi), a long-lasting single-dose RSV monoclonal antibody, has demonstrated efficacy in preventing RSV-related ALRTI in both preterm and term infants, maintaining protection five months after administration [11,14]. Additionally, RSVpreF, a maternal vaccine (MI) known as Abrysvo™ (Pfizer), has proven effective in providing protection to infants [13]. This protection is achieved through the transplacental transfer of antibodies during the first six months of life [13]. Both interventions have recently received regulatory approval from various agencies worldwide, and are beginning to be introduced into national immunization programmes [15,16].

To make informed recommendations about the use of RSV mAb and RSV maternal vaccine in Argentina, national decision-makers require evidence on their potential impact and cost-effectiveness [17]. The aim of this study is to estimate the potential costs and consequences of each intervention using the best input data currently available for the Argentine context. The study can also be used to identify the maximum dose price that could be acceptable [[18], [19], [20]].

## 2 Methods

### 2.1 Decision-support model

We used UNIVAC (version 1.5.21), a static cohort model with a finely disaggregated age structure (weeks of age < 5 years) to estimate the potential impact and cost-effectiveness of implementing RSV mAb or maternal RSV vaccine in Argentina over a ten-year period (2025–2034) [18,20]. The methods for evaluating RSV interventions in UNIVAC are described in detail elsewhere [18,19]. In brief, for each birth cohort, the numbers of life-years experienced between birth and age 5.0 years are multiplied by the rates of RSV disease cases and deaths among individuals under the age of 5 to estimate the expected number of cases, deaths, and Disability Adjusted Life Years (DALYs) that will occur with and without RSV interventions over the lifetimes of each birth cohort [18]. The model also calculates the costs of RSV prevention strategies and the healthcare expenses associated treating RSV disease cases with and without RSV intervention strategies [18]. In the base case scenario of the model RSV mAb or maternal RSV vaccine were assumed to be administered year-round, but we also ran alternative what-if scenarios using seasonal approaches [18,20].

The primary outcome measure was the cost per DALY averted from a societal perspective, which considered all lifetime costs and benefits accumulated over the ten birth cohorts (2025–2034) [18]. All future costs and health benefits were discounted at a rate of 3% per year, and all costs are expressed in 2023 US$ currency exchange rate to ARS$ = 0·0028 [21]. We compared each option to no RSV intervention and to each other [13,14]. We used a willingness-to-pay (WTP) threshold for Argentina of 0.9 times the Gross Domestic Product (GDP) per capita based on the recommendation of the Argentinean health technology assessment commission [22]. However, since the threshold has not been formally established in Argentina, two additional thresholds were included (i.e., 0·6 and 1·2 times the GDP/capita) [22]. We assumed the GDP per capita was US$ 12,345 for Argentina, based on estimates from the World Bank for the year 2022 [23].

### 2.2 RSV disease burden

Disease event rates related to RSV in children aged <5 years in Argentina were determined based on an ongoing cohort study conducted within a catchment population of 870,000 children aged <5 years in the Buenos Aires Metropolitan Region (see supplementary material). This extensive data collection spans from 2018 to 2023. Supplementary Fig. 1 illustrates the age distribution of RSV hospitalizations in this population. Non-severe and severe outpatient visits data were obtained from the outpatient clinics information system, while hospitalization rates were acquired through bed occupancy data specific to RSV-related diseases. RSV mortality rates encompassed both in-hospital mortality and community mortality. The data was used to derive rates of RSV clinic visits, hospital admissions and deaths per 100,000 per year, in children aged <5 years. The rates were also compared to other published sources (see supplementary material) [4,6].

### 2.3 Healthcare costs

In the base case scenario, a societal perspective was used to derive the average cost per RSV clinic visit and RSV hospital admission. Costs included direct medical costs (e.g. drugs, diagnostics), direct non-medical costs (e.g. transportation) and indirect costs (e.g. opportunity costs of lost income). The costs were informed by the published literature and an ongoing cost of illness study on RSV in Argentina [9,24]. The costs are summarized in Table 1. More detail on the data sources is provided in the supplementary appendix.

**Table 1:** Input parameters. Cost-effectiveness of RSV prevention strategies in Argentina for 10 birth cohorts. Period 2025–2034.

**Input parameter**	**Value**	**Uncertainty range**	**Source**
**RSV disease event rate per 100,000 per year (<5 yrs)**			
RSV (non-severe) cases	12,497	3757- 17,117	Surveillance data of 4 argentinean hospitals from 2018 to 2019 (Suppl. appendix)
RSV (non-severe) visits	8991	2703-12,314	
RSV (severe) cases	1690	1026-2202	
RSV clinic visits	1444	968–1721	
RSV hospital admissions	1010	677–1203	
RSV deaths	15	7–22	
**DALY weights**			
RSV non-severe cases	5.10%	3.20–7.40%	Bazargani et al. (19)
RSV severe cases	13.30%	8.80–19.00%	Bazargani et al. (19)
**Duration of illness (days)**			
RSV non-severe cases	2.70	1.90–3.80	Surveillance data of 4 argentinean hospitals from 2018 to 2019 (Suppl. appendix)
RSV severe cases	4.10	2.70–5.70	
**Disease treatment costs (US$) [Government perspective]**			
RSV (non-severe) clinic visits	$ 31.20	28.04–45.66	Surveillance data of 4 argentinean hospitals from 2018 to 2019 (Suppl. appendix)
RSV (severe) clinic visits	$ 74.61	64.26–76.26	Surveillance data of 4 argentinean hospitals from 2018 to 2019 (Suppl. appendix)
RSV hospital admissions	$ 587.79	577.94–695.33	Dvorkin et al. (8)
**Disease treatment costs (US$) [Societal perspective]**			
RSV (non-severe) clinic visits	$ 36.09	32.31–51.16	Surveillance data of 4 argentinean hospitals from 2018 to 2019 (Suppl. appendix)
RSV (severe) clinic visits	$ 79.50	68.53–81.76	Surveillance data of 4 argentinean hospitals from 2018 to 2019 (Suppl. appendix)
RSV hospital admissions	$ 636.69	577.95–695.83	Dvorkin et al. (8)
**Efficacy of RSV vaccine (maternal)**			
Program coverage	74.80%	57.80–98.60%	Arg.Min. Health(20) Kampmann et al.(15) Kampmann et al.(15) Kampmann et al.(15)
Efficacy (RSV non-severe cases)	48.58%	31–58.90%	
Efficacy (RSV severe cases)	69.40%	44.30–84.10%	
Duration of protection (months)	6	3–7	
**Efficacy of RSV mAb (infant)**			
Program coverage	80.90%	66.10–94.10%	Arg.Min. Health(20) Muller et al.(10) Muller et al.(10) Muller et al.(10)
Efficacy (RSV non-severe cases)	62.58%	41.60–73.16%	
Efficacy (RSV severe cases)	76.8%	49.40–89.40%	
Duration of protection (months)	5	4–6	
**Program costs (US$) for both maternal vaccine and infant mAb**			
Wastage of doses (%)	5.00%	2.00–8.00%	Assumption
Wastage of syringes (%)	5.00%	2.00–8.00%	Assumption
Wastage of safety boxes (%)	5.00%	2.00–8.00%	Assumption
Price per dose	$ 3.30	–	UNICEF Sup Div(23)
Price per syringe (US$)	$ 0.0278	–	UNICEF Sup Div(23)
Price of safety boxes per dose (US$)	$ 0.0121	–	Debellut et al.(26)
International handling (% of price per dose)	1.40%	–	UNICEF Sup. Cat.(24)
International delivery (% of price per dose)	6.00%	2.00–15.00%	UNICEF Sup.Cat(24)
Incremental health system cost per dose (US$)	$ 2,02	–	ICAN(25)

### 2.4 RSV prevention strategy costs

Intervention cost inputs are summarized in Table 1 [[25], [26], [27]]. The cost of RSV mAb remains uncertain in Argentina, so we developed three different price-per-dose (PPD) scenarios with US $50 per dose for the base-case analysis as well as US $25 and US $100 per dose. We obtained the costs for international handling and delivery, safety boxes, and syringes from relevant sources [20,28]. We assumed a 5% wastage rate for the doses, syringes, and safety boxes (Table 1) and converted this into a wastage factor [1/ (1- % wastage)], which was multiplied by the expected number of doses needed to achieve the desired level of coverage. For both interventions we assumed an additional cost of US $2·02 per dose to account for costs associated with program delivery including supplementary training, transportation, and enhancing cold chain capacity. As no empirical data is available from Argentina on the program delivery costs of these interventions, we have relied on existing literature and the Immunization Delivery Cost Catalogue (IDCC) repository to derive our estimates.

### 2.5 Impact of RSV prevention strategies

We assumed a maternal vaccination coverage of 74%, based on the flu vaccine coverage rate reported in the Argentine literature [29]. We had insufficient data to allow estimates of coverage by week of gestation, so assumed one coverage estimate applied to all pregnant women, with protection initiated from birth. However, to address potential uncertainty we varied it in probabilistic and deterministic sensitivity analysis. We also assumed that mAb coverage could reach 80·9% according to coverage reported for Bacille Calmette-Guérin (BCG) vaccination in Argentina [29].

We used the reported results from recent efficacy trials for RSV mAb and maternal RSV vaccine. Efficacy against all severe RSV outcomes was assumed to be 76·8% (95% CI, 49·4 to 89·4) for 5 months for RSV mAb and 69·4% (95% CI, 44·3 to 84·1) for 6 months for maternal RSV vaccine (see Table 1) [13,14]. Efficacy against all non-severe outcomes was assumed to be 62·6% (95% CI 41·6 to 71·2) for RSV mab and 48·6% (95% CI 31 to 58·9) for maternal RSV vaccine based on efficacy against medically attended RSV [10,12].

### 2.6 Uncertainty analysis

We varied each parameter by ±10% in order to assess its influence on the cost-effectiveness results [30]. We also ran probabilistic sensitivity analyses (PSA) to estimate the range of uncertainty around each of the three fixed price scenarios (US$ 25, 50 and 100). For each price scenario we ran 1000 probabilistic simulations. For each input parameter we specified a mid-value, range and simple PERT-Beta distribution [18,20]. Finally, we ran a range of deterministic (what-if) scenarios including: (i) higher/lower coverage of both interventions; (ii) reduced duration of maternal vaccination protection with increased efficacy: 81·8% for 3 months [13]; (iii) gradual waning for RSV mAb and maternal RSV vaccine protection assuming a gamma curve with an initial maximum efficacy and a gradual reduction in protection reaching zero at 12 months [18]; and, (iv) seasonal dose administration for RSV mAb and maternal RSV vaccine. This evaluation utilized data from 1893 RSV-positive hospital admissions recorded between 2018 and 2019 in children under 5 years and estimated the level of protection given their date of birth and date of hospital admission. We explored a range of potential months of birth that could be targeted for protection using either RSV mAb and maternal RSV vaccine [4,6], and compared each combination to the expected health impact (percent reduction in RSV hospital admissions) and dose efficiency (doses required to achieve a 1% reduction in RSV hospital admissions) of a year-round strategy.

## 3 Results

### 3.1 Lifetime healthcare effects

We initially examined and compared the lifetime costs and consequences of implementing three policy options (no RSV intervention, RSV mAb, and maternal RSV vaccine) in Argentina over a 10-year period (2025–2034) for a price per dose of US$50 for each strategy.

Implementing either RSV prevention strategy could prevent approximately 500,000 RSV disease cases (RSV mAb: 620,601 vs. maternal RSV vaccine: 431,589), a substantial number of RSV clinic visits (RSV mAb: 461,609 vs. maternal RSV vaccine:324,429), over 50,000 RSV hospital admissions (RSV mAb: 66,921 vs. maternal RSV vaccine: 61,633), and over 1000 RSV deaths (RSV mAb: 1451 vs. maternal RSV vaccine: 1313). Both strategies were estimated to prevent >25% of RSV deaths in children aged under 5 years, and ∼ 30% in children aged <6 months (Supplementary Fig. 2 and Table 2). Both interventions would avert over 35,000 DALYs for the entire analyzed period (RSV mAb: 38,791 vs maternal RSV vaccine: 35,050).

**Table 2:** Lifetime costs and effects of maternal vaccination ($50/dose, 69% efficacy, 6 months protection) and infant mAb ($50/dose, 75% efficacy, 5 months protection) in Argentina. Period 2025–2034.

	**No RSV intervention**	**Infant monoclonal antibody (RSV mAb)**	**Maternal** **vaccine (maternal RSVvaccine)**
**Lifetime costs and effects**			
Non-severe RSV cases <5 yrs	3,856,021	3,347,397	3,527,560
Non-severe RSV clinic visits <5 yrs	2,774,225	2,408,294	2,537,912
Severe RSV cases <5 yrs	521,459	409,482	418,331
Severe RSV clinic visits <5 yrs	445,554	349,877	357,438
Severe RSV hospital admissions <5 yrs	311,641	244,720	250,008
Severe RSV deaths <5 yrs	4628	3178	3315
DALYs (discounted⁎)	124,241	85,450	89,191
RSV strategy costs (discounted⁎)	–	$ 259,847,274	$ 240,981,575
Government healthcare costs (discounted⁎)	$ 257,459,185	$ 207,333,754	$ 214,014,047
Societal healthcare costs (discounted⁎)	$ 283,757,121	$ 228,843,772	$ 236,328,668
**Differences (comparator = no vaccine)**			
Non-severe RSV cases <5 yrs	–	−508,624	−328,461
Non-severe RSV clinic visits <5 yrs	–	−365,931	−236,313
Severe RSV cases <5 yrs	–	−111,977	−103,128
Severe RSV clinic visits <5 yrs	–	−95,678	−88,116
Severe RSV hospital admissions <5 yrs	–	−66,921	−61,633
Severe RSV deaths <5 yrs		−1451	−1313
Percent reduction in severe RSV deaths <5 yrs	–	−31,30%	−28,37%
DALYs (discounted⁎)	–	−37,581	−35,050
RSV strategy costs (discounted⁎)	–	$ 259,847,274	$ 240,981,575
Government healthcare costs (discounted⁎)		-$ 50,125,431	-$ 43,445,138
Societal healthcare costs (discounted⁎)	–	-$ 54,913,349	-$ 47,428,453
**Cost (US$) per DALY averted (comparator = no vaccine)**			
*Government cost perspective*			
Net cost (strategy cost – averted costs) (discounted⁎)		$ 209,721,843	$ 197,536,437
DALYs averted (discounted⁎)	–	38,791	35,050
Cost per DALY averted (discounted⁎)	–	$ 5407	$ 5636
*Societal cost perspective*			
Net cost (strategy cost – averted costs) (discounted⁎)	–	$ 204,933,925	$ 193,553,122
DALYs averted (discounted⁎)	–	38,791	35,050
Cost per DALY averted (discounted⁎)	–	$ 5283⁎⁎	$ 5522⁎⁎

⁎ Future costs/effects were discounted at a rate of 3% per year.

⁎⁎ Very subtle changes in parameters (price, efficacy etc) could lead to a different rank order of cost-effectiveness, hence presenting the cost-effectiveness of both interventions (RSV mAb and maternal RSV vaccine) compared to nothing, rather than to each other directly.

### 3.2 Economic impact and cost-effectiveness

A ten-year program of RSV mAb year-round administration was estimated to cost approximately US$ 259 million after discounting for present values. The equivalent program cost for maternal RSV vaccine was estimated to be approximately US$ 240 million reflecting the lower intervention coverage assumptions for maternal vaccination. At least 25% of this cost would be offset by public sector healthcare cost savings, with slightly higher savings from a societal perspective. From the societal perspective, both interventions will generate healthcare costs savings (e.g., avoided hospitalizations) of approximately US$ 50 million (RSV mAb: US$ 54,913,349 vs. maternal RSV vaccine: US$ 47,428,453).

From a societal perspective, both interventions demonstrated similar cost-effectiveness when compared separately to no intervention (US$5283 [95%CI US$5203 to US$5363] and US$5522 [95%CI US$5427 to US$5617] per DALY averted for RSV mAb and maternal RSV vaccine, respectively) (Table 2).

### 3.3 Uncertainty analysis

**Fig. 1:**
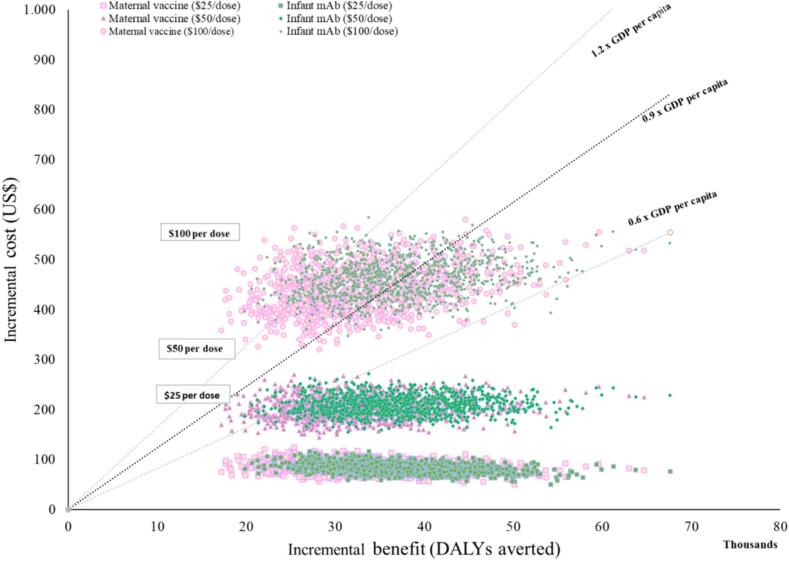
Probabilistic clouds showing the incremental cost (US$) and benefit (DALYs averted) for both RSV prevention strategies compared to no intervention with three different dose prices ($25, $50 and $100) and three different WTP thresholds (1,2; 0.9 and 0.6 GDP per capita).

**Fig. 2:**
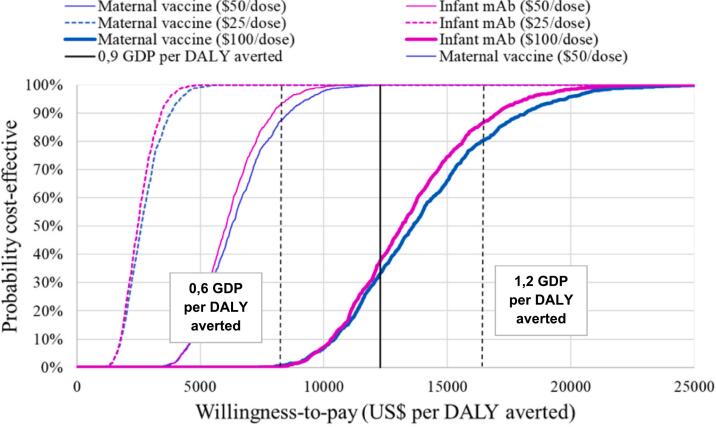
Probability that each RSV prevention strategy will be cost-effective compared to no intervention, at different willingness-to-pay thresholds.

The probabilistic clouds of uncertainty around the cost-effectiveness results were similar for both strategies (Fig. 1). Per the reference WTP threshold of 0·9 GDP/capita, both strategies were cost-effective in 100% of the simulated scenarios at baseline PPD. Should the WTP threshold be increased to 1·2 GDP/capita, then a PPD of US$100 would have approximately 80% probability of being cost-effective for both interventions (Fig. 2). Supplementary Tables 1 and 2 provide details on clinical and economic outcomes for the US$25 and US$100 scenarios, respectively.

When analyzing the results for a single cohort (one year), we could observe that interventions would prevent approximately 30,000 clinic visits (RSV mAb: 46,329 vs. maternal RSV vaccine: 32,481), 6000 hospitalizations (RSV mAb: 6716 vs. maternal RSV vaccine: 6186) and over 100 deaths (RSV mAb: 146 vs. maternal RSV vaccine: 132). For this single cohort, the cost-effectiveness ratio would be US$5288 per DALY averted for RSV mAb and US$5532 for maternal RSV vaccine. Full results for the single cohort are shown in Supplementary Table 3.

**Fig. 3:**
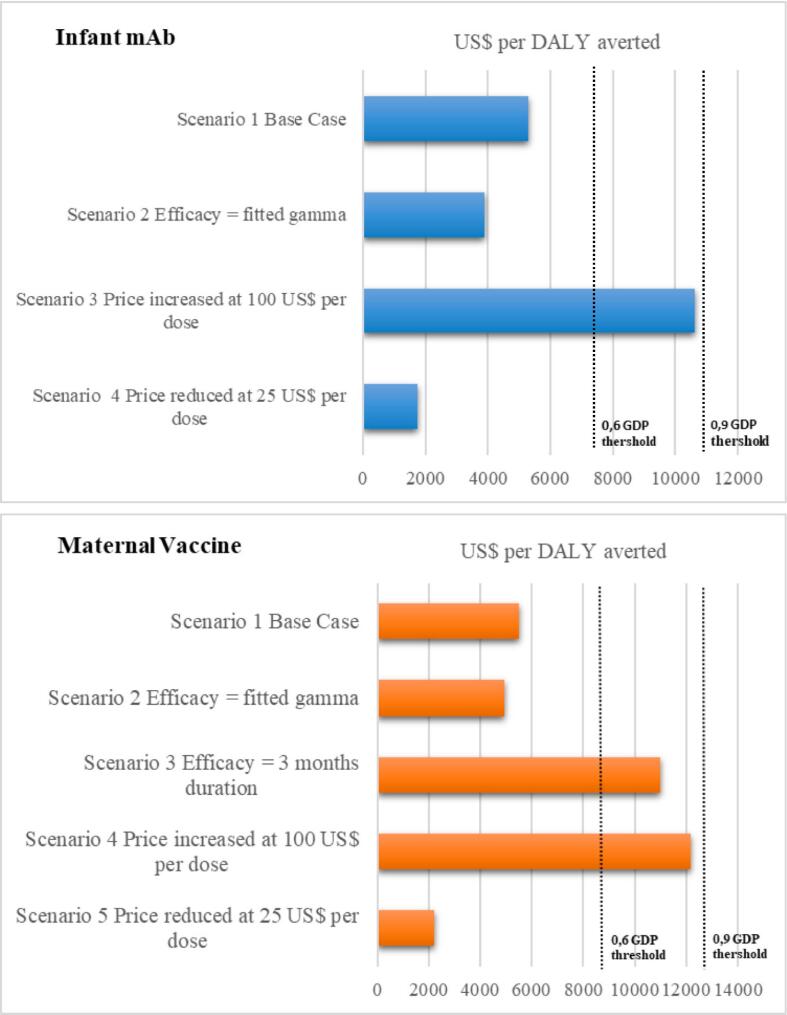
Cost (US$) per DALY averted for alternative deterministic scenarios (comparator = no RSV intervention strategy). Societal perspective.

**Fig. 4:**
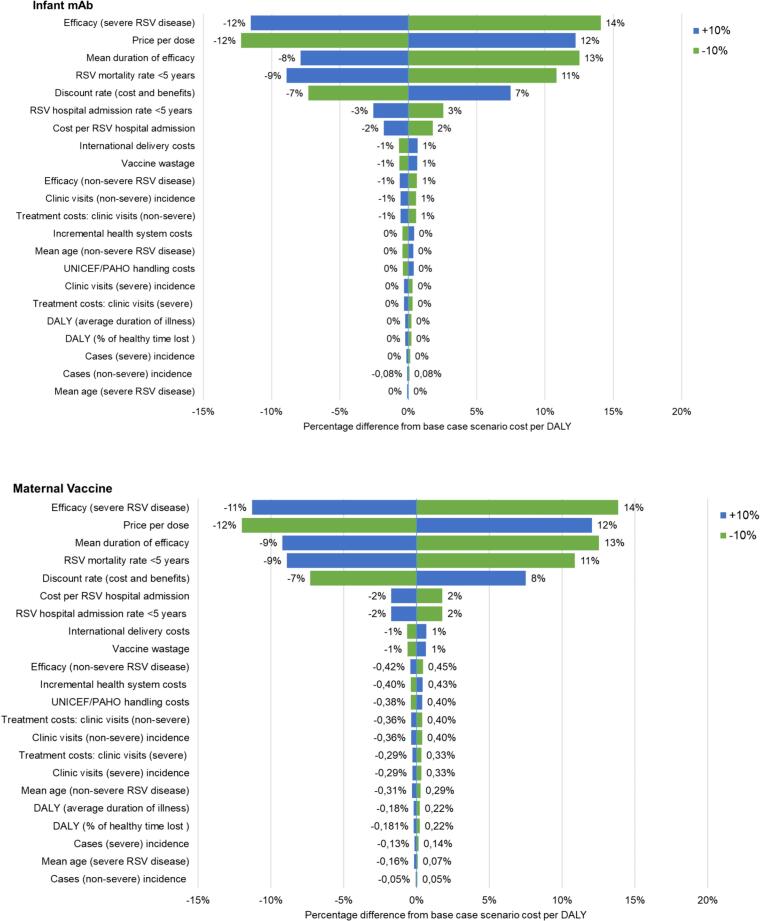
Deterministic sensitivity analysis on the incremental cost effectiveness ratio (ICER) for RSVstrategies in Argentina. Values at the end of each variable line show base [min; max].

Assuming alternative efficacy and waning assumptions led to variations in the cost-effectiveness ratio as presented in Fig. 3. For both interventions, a waning effect would make the cost per DALY averted more favorable. The tornado diagram (Fig. 4) illustrates that efficacy, PPD, efficacy duration, and RSV mortality rate in children under the age of 5 had the greatest impact on the cost per DALY averted for both interventions. Reducing the baseline intervention coverage (RSV mAb 80·9% and maternal RSV vaccine 74%) to 50% resulted in a higher number of RSV hospital admissions and deaths (10.05% increase for RSV mAb and 8.17% increase for maternal RSV vaccine). (Supplementary Table 3).

**Fig. 5:**
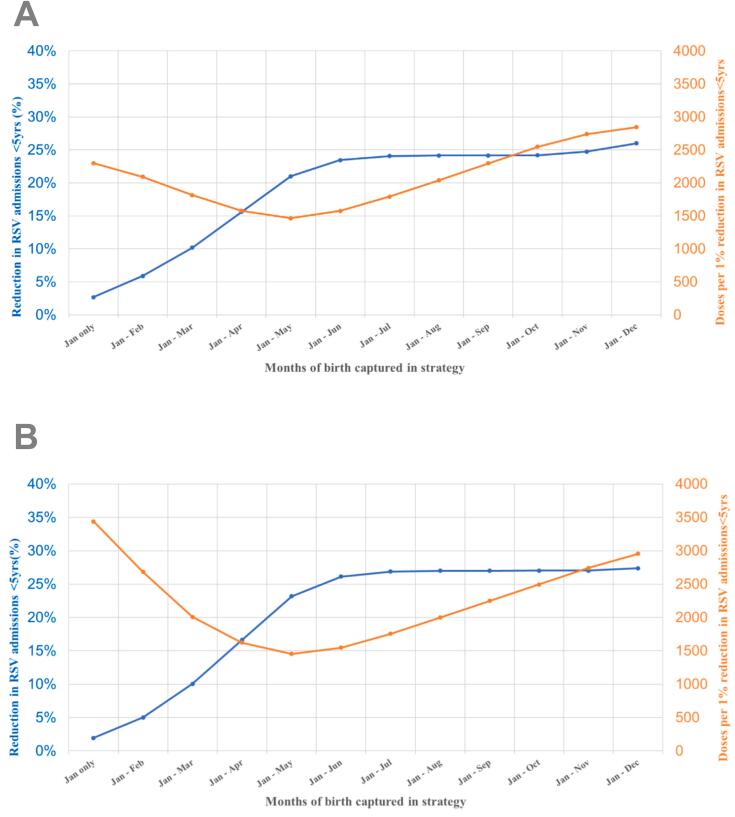
Analysis of clinical impact and cost-effectiveness with different seasonal coverage strategies. Reduction of admissions and doses for each 1% reduction. Efficacy for fixed duration^⁎^. [A] RSV mAb, [B] Maternal RSV Vaccine. * Figures for waning efficacy can be found in the Appendix

Considering Argentina's seasonal burden of RSV disease with much of the burden occurring from May to September, we explored a range of seasonal rather than year-round strategies [4,5]. Data acquisition for this analysis is explained in the Supplementary Appendix. This involved varying the months of birth targeted for protection [5]. Assuming a 6-month strategy (i.e. from January to June) and efficacy for fixed duration, maternal RSV vaccine exhibited a 11·5% reduction in impact and a 45% improvement in dose efficiency (Supplementary Table 4 and Fig. 5a) when compared to a year-round immunization program. In comparison, RSV mAb demonstrated a 3·7% reduction in impact and a 48% increase in dose efficiency when compared to a year-round immunization program (Supplementary Table 5 and Figure 5B). Results assuming that efficacy wanes gradually are shown in the supplementary material.

## 4 Discussion

We evaluated the immunization programs for RSV disease in Argentina, involving the administration of RSV mAb to young infants and the maternal RSV vaccine to pregnant women [13,14]. Our estimates indicate that both immunizations will reduce RSV total cases, hospital admissions, and deaths.

Employing a year-round approach in Argentina, both the maternal immunization RSVpreF (Abrysvo™) and the RSV mAb Nirsevimab (Beyfortus™) were cost-effective in the deterministic and probabilistic sensitivity analyses at a per-dose cost of US$50 and a willingness-to-pay (WTP) threshold of 90% of the country's GDP/capita. Both interventions demonstrated similar performance in the deterministic analysis. However, cost-effectiveness is heavily dependent on the prices of the strategies, and intervention prices are currently uncertain in Argentina.

Similarly to RSV, previous implementations of other vaccine strategies in Argentina, such as the pneumococcal conjugate vaccine (PCV) or rotavirus vaccine, have demonstrated cost-effectiveness in infants [31,32]. In this context, both strategies could be considered feasible for implementation, depending on the available health budget and the prioritization of health strategies by the country. Moreover, both products have received approval from the National Administration of Drugs, Food and Medical Devices (ANMAT) in Argentina, with the maternal immunization being implemented since 2024 [16]. However, in the incorporation of this vaccine, it was not made explicit whether economic criteria were used to decide its inclusion in the immunization schedule [33]. Cite Considering the eventual creation of a binding health technology assessment agency in the country, this information may be useful for future decisions. In this context, it is crucial to emphasize the importance of continuously monitoring the ongoing impact and safety, and if necessary, updating the cost-effectiveness using improved post-licensure data.

When analyzing the net government costs of introducing these interventions, we found that they would each of them would represent approximately 0·34% of total public health expenditure and 6·4% of the public budget for immunization per year for Argentina [21,22,34].

Considering the clear homogenous seasonal circulation pattern of RSV in Argentina, we extended the analysis to assess the impact and cost-effectiveness within the context of potential seasonal immunization programs [4,6,17,35,36]. We found that a seasonal strategy from January to July could improve the ICER with a small detrimental effect on overall impact. Specifically, a seasonal strategy would fail to avert one in ten cases that would have been averted by a year-round strategy but is more efficient in the cost to prevent each case. The seasonal vaccination strategy draws on the existing influenza vaccination approach in the country as a potential model [37]. However, ethical considerations regarding equitable access to both interventions should be thoroughly examined, especially considering a potential reduction in the overall impact of the program due to a seasonal strategy [38]. Additionally, there may be additional costs associated with implementing this strategy (e.g., awareness campaigns, and training) that were not included in our analysis.

This is one of the first uses for the UNIVAC RSV module. Regarding our assumptions, it is important to highlight some aspects. Both RSV mAb and maternal RSVvaccine are expected to prevent disease in the first 6 months of life, but we estimate RSV disease burden up to age 5.0 years for completeness; this does not affect estimates of cost-effectiveness but does allow interpretation of the under-five RSV burden that is not prevented by these interventions. Additionally, we chose a static model because there is limited evidence that the current interventions will interrupt transmission. Finally, we evaluated a ten-year intervention program (annual implementation of RSV interventions for ten successive birth cohorts) as this allows estimates of the potential annual intervention budget impact, and allows some parameters to vary with time e.g., the counterfactual rate of RSV mortality may decline, even in the absence of new pharmaceutical interventions.

A prior study in Vietnam also used the same model [20]; however, our investigation differs in different input parameters and incorporates a seasonal analysis. Despite these distinctions, our overall conclusions align with theirs, emphasizing that efficacy and price play crucial roles in determining cost-effectiveness [20].

Our study has several strengths. First, our study uses good quality data on the burden and cost of RSV illness in the Argentine context [4,6,9]. Second, the transparent UNIVAC decision support model allows data inputs to be shared and interrogated by national stakeholders and could easily be updated as new data emerges [[18], [19], [20]]. Finally, our cost-effectiveness conclusions were robust to a range of uncertain what-if scenarios, including possible seasonal immunization programs [17].

The study had certain limitations and challenges. Estimating coverage rates for RSV prevention strategies can be complex, especially given the post-COVID-19 pandemic landscape with fluctuations in vaccine coverage rates [38]. These variations are particularly notable in other maternal vaccination programs within the country [29]. Accuracy hinges on factors such as public acceptance and potential shifts in healthcare dynamics. Our analysis does not account for any indirect effects but there is limited evidence that the current interventions will interrupt transmission. We did not include any additional program costs that may be associated with targeting mothers (and infants) that would be eligible for a seasonal strategy. We also did not consider the potential combined use of both strategies i.e. targeting RSV mAb to infants not previously protected through maternal vaccination.

In the absence of a pediatric vaccine, careful consideration of effective implementation strategies for passive immunizations is crucial, with an eye on their long-term impact [17]. One potential scenario would involve the implementation of two combined preventive strategies, similar to approaches used for influenza or pertussis, at a cost affordable for most LMICs [29]. Another alternative could be the combination of strategies targeted at subpopulations with a high risk of severe disease. Conducting combined implementation studies to gather real-life evidence could pave the way for developing precise and cost-effective usage policies in each region. Lastly, the implementation of one or both preventive measures will enable a comprehensive assessment of their real-life impact, particularly in terms of hospital and community mortality, particularly in LMICs.Recent data from Spain on the implementation of RSV mAb have been conclusive, demonstrating a significant reduction in hospitalizations due to RSV in infants and children in the last RSV season [15].

While various variables influenced the effectiveness of the interventions, such as PPD, efficacy, mortality, and health costs, we recognize that the pricing of the products would play a crucial role in determining the cost-effectiveness balance for both interventions. Nevertheless, these advancements hold promise for combating severe RSV disease in young infants. The considerations of accessibility and cost underscore the importance of ensuring equitable access, particularly in resource-constrained settings within the country and the region [17,38].

In conclusion, our preliminary assessment suggests that either RSV mAb and maternal RSV vaccine could be cost-effective in Argentina, contingent on suitable pricing. Furthermore, these interventions have the potential to reduce clinic visits, hospitalizations, and deaths, alleviating the burden on the Argentinean healthcare system. A six-month seasonal immunization strategy appears to be a viable and cost-effective approach, aligning with the viral circulation patterns in Argentina [4,6]. Our analysis and findings provide valuable insights for decision-makers, offering a foundation for informed policy recommendations regarding the adoption of these interventions in the region.

## Funding

This work was supported by the 10.13039/100000865Bill & Melinda Gates Foundation (Grant Number INV-007610). Under the grant conditions of the foundation, a Creative Commons Attribution 4.0 generic License has already been assigned to the Author Accepted Manuscript version that might arise from this submission. The findings and conclusions contained within are those of the authors and do not necessarily reflect the positions or policies of the Bill & Melinda Gates Foundation.

## CRediT authorship contribution statement

**Gonzalo Guiñazú:** Writing – review & editing, Writing – original draft, Validation, Project administration, Methodology, Investigation, Formal analysis, Data curation, Conceptualization. **Julia Dvorkin:** Writing – review & editing, Writing – original draft, Methodology, Data curation, Conceptualization. **Sarwat Mahmud:** Writing – review & editing. **Ranju Baral:** Writing – review & editing, Resources. **Clint Pecenka:** Writing – review & editing, Conceptualization. **Romina Libster:** Writing – review & editing. **Andrew Clark:** Writing – review & editing, Writing – original draft, Methodology, Formal analysis, Conceptualization. **Mauricio T. Caballero:** Writing – review & editing, Writing – original draft, Supervision, Project administration, Methodology, Investigation, Funding acquisition, Formal analysis, Data curation, Conceptualization.

## Declaration of competing interest

The authors declare the following financial interests/personal relationships which may be considered as potential competing interests: Mauricio Caballero reports financial support was provided by Bill & Melinda Gates Foundation. If there are other authors, they declare that they have no known competing financial interests or personal relationships that could have appeared to influence the work reported in this paper.

## Data Availability

Data will be made available on request.

## Appendix A Supplementary data

Supplementary material: Supplementary Appendix Image 1
