# Corrigendum to “Evaluation of the potential impact and cost-effectiveness of respiratory syncytial virus (RSV) prevention strategies for infants in Argentina” [Vaccine. Volume 42, Issue 23, 3 October 2024, 126234]

**DOI:** 10.1016/j.vaccine.2024.126402

**Published:** 2025-01-12

**Authors:** Gonzalo Guiñazú, Julia Dvorkin, Sarwat Mahmud, Ranju Baral, Clint Pecenka, Romina Libster, Andrew Clark, Mauricio T. Caballero

**Affiliations:** a Centro INFANT de Medicina Traslacional (CIMET), Escuela de Bio y Nanotecnología (EByN), Universidad Nacional de San Martín (UNSAM), Buenos Aires, Argentina; b Fundación INFANT, Buenos Aires, Argentina; c Consejo Nacional de Investigaciones Científicas y Técnicas (CONICET), Argentina; d Department of Health Services Research and Policy, Faculty of Public Health and Policy, London School of Hygiene and Tropical Medicine, London, UK; e Center for Vaccine Innovation and Access, PATH, Seattle, WA, USA

The authors regret < We have found some typos in the online publication, and we would kindly like to ask you to correct them.

1 Abstract – “Either strategy (RSV mAb or maternal RSV vaccine) could prevent >25% of RSV deaths aged <5 years and ∼30% aged <6 months (the age group where most intervention impact occurs).” should be “Either strategy (RSV mAb or maternal RSV vaccine) could prevent >25% of RSV deaths aged <5 years and >50% of RSV deaths aged <6 months (the age group where most intervention impact occurs).”

2 Section 3.1 - “Both strategies were estimated to prevent >25% of RSV deaths in children aged under 5 years, and ∼ 30% in children aged <6 months” should be “Both strategies were estimated to prevent >25% of RSV deaths in children aged under 5 years, and >50% of RSV deaths in children aged <6 months”.

3 In table 1 “Price per dose” is showing $3.30 should be $50>.

The authors would like to apologise for any inconvenience caused.

