# Supplementary material for: Evaluation of the potential impact and cost-effectiveness of respiratory syncytial virus (RSV) prevention strategies for infants in Argentina
Source: Vaccine. 2024 Oct 3;42(23):None. doi: 10.1016/j.vaccine.2024.126234 (PMC11413482; doi:10.1016/j.vaccine.2024.126234)
Supplement: Supplementary file 1 — Supplementary material: Supplementary Appendix [file mmc1.docx]

**SUPPLEMENTARY APPENDIX**

**Evaluation of the Impact and Cost-Effectiveness of Respiratory Syncytial Virus Prevention Strategies for Infants in Argentina. Supplementary appendix**

Gonzalo Guiñazú^1^; Julia Dvorkin^1,2,3^; Sarwat Mahmud^4^; Emiliano Sosa^1,2^; Elizabeth Vodicka^5^, Ranju Baral^5^, Clint Pecenka^5^, Romina Libster^2^, Andrew Clark^6*^, and Mauricio T. Caballero^1,2,3*^.

Affiliations: 1Centro INFANT de Medicina Traslacional (CIMET), Escuela de Bio y Nanotecnología (EByN), Universidad Nacional de San Martín (UNSAM), Buenos Aires, Argentina. 2Fundación INFANT, Buenos Aires, Argentina. 3Consejo Nacional de Investigaciones Científicas y Técnicas (CONICET), Argentina. 4Department of Health Services Research and Policy, Faculty of Public Health and Policy, London School of Hygiene and Tropical Medicine, London, UK. 5Center for Vaccine Innovation and Access, PATH, Seattle, Washington, USA 6Department of Health Services Research and Policy, Faculty of Public Health and Policy, London School of Hygiene and Tropical Medicine, London, UK.

**Table of contents**

| Title | 1 |
| --- | --- |
| List of authors and affiliations | 1 |
| Table of contents | 2 |
| Data acquisition for epidemiological and cost parameters description | 3-5 |
| Supplementary Figure 1 | 6 |
| Supplementary Table 1 | 7 |
| Supplementary Figure 2 | 8 |
| Supplementary Table 2 | 9 |
| Supplementary Table 3 | 10 |
| Supplementary Figure 3 | 11 |
| Supplementary Table 3 | 12 |
| Supplementary Table 4 | 13 |
| Seasonal approach. Efficacy waning | 14 |
| Supplementary Figure 4 | 15 |
| References | 16 |

**Data acquisition for epidemiological and cost parameters**

Leveraging our Infant-RSV Network in the Buenos Aires Metropolitan Region (BAMR), we conducted active surveillance of viral infections from 2011 to 2023. Particularly for the purpose of this analysis we utilized data from 2018 to 2023 from 4 tertiary hospitals from the Network. The BAMR, in which the INFANT-RSV Network is located, is an urban area that includes the City of Buenos Aires and 40 surrounding counties of the Buenos Aires Province and the City of Buenos Aires. It covers 13,285 km^2^ and has 14,800,000 inhabitants, representing 37% of Argentina´s total population. The INFANT-RSV Network reaches an estimated population of ~870,000 infants and children younger than 5 years of age. Information was obtained and uploaded in research forms and files from medical records and registrations across outpatient clinics, emergency rooms, wards, and intensive care units. Additionally, open access Government statistics were analyzed for this purpose. All disease event rates in children under the age of 5 were calculated per 100,000 per year.

Definitions:

*RSV non-severe disease, cases*: Patients who visit outpatient clinics, ER, and hospitals during any mild acute respiratory disease due to RSV with home discharge from the visit, plus those estimated no concurring to any healthcare provider as calculated by Li et al. (1)

*RSV non-severe, visits:* Patients who visit outpatient clinics, ER, and hospitals during any mild acute respiratory disease due to RSV with home discharge from the visit. RSV severe disease, cases: Patients who visit outpatient clinics, ER, and hospitals during an acute lower respiratory infection due to RSV, defined as: the presence of at least one manifestation of lower respiratory tract infection signs (cough, nasal flaring, indrawing of the lower chest wall, subcostal retractions, stridor, rales, rhonchi, wheezing, crackles or crepitations, or observed apnea) plus hypoxemia (peripheral oxygen saturation <95% at FiO2=0.21) or tachypnea (=70 breaths per minute from 0 to 59 days of age and =60 breaths per minute at 60 days of age or older) plus those who die at home plus those estimated no concurring to any healthcare provider.

*RSV severe disease, visits*: Patients who visit outpatient clinics, ER, and hospitals during an acute lower respiratory infection due to RSV, defined as above and was discharged before 24 hours of admission. RSV severe disease, hospitalization: Patients who were hospitalized in the ER department, regular ward and PICU during an acute lower respiratory infection due to RSV, defined as above.

*RSV severe disease, deaths:* All patients who die due to RSV at home or hospitals based on estimations published on Caballero et al. (2) and Geoghegan et al. (3)

Cost of acute RSV disease

We used information from a prospective cost study approved for publication in Dvorkin et al (4). Briefly, in this study we assessed direct medical costs associated with ALRTI due to RSV. Direct medical cost data collected included medical procedures, medications, laboratory tests, consumables, and bed nights, among others. These costs, as well as non-medical overhead costs, were obtained from hospital administrators, financial officers, pharmacy administrators, supply chain managers, logisticians/procurement staff, laboratory managers, and others with knowledge about hospital finances and/or itemized unit costs. Additionally, we assessed resource utilization via secondary data analysis of the existing study database. The database contains information on healthcare resource utilization, RSV diagnosis, and demographic information from patients experiencing RSV-ALRTI. Healthcare utilization data was pulled for each child to identify resources used during the child’s course of treatment in the facility. If any data gaps were identified during the secondary analysis, we conducted a medical record review for any enrolled children for whom data is not currently available through our study dataset.

For indirect costs and non-medical cost our research staff interviewed parents and families to capture out-of-pocket costs, salary, and other extra expenses related to ALRTI disease and uploaded in research forms properly.

**Supplementary Figure 1. Age distribution of RSV disease cases in Argentina**


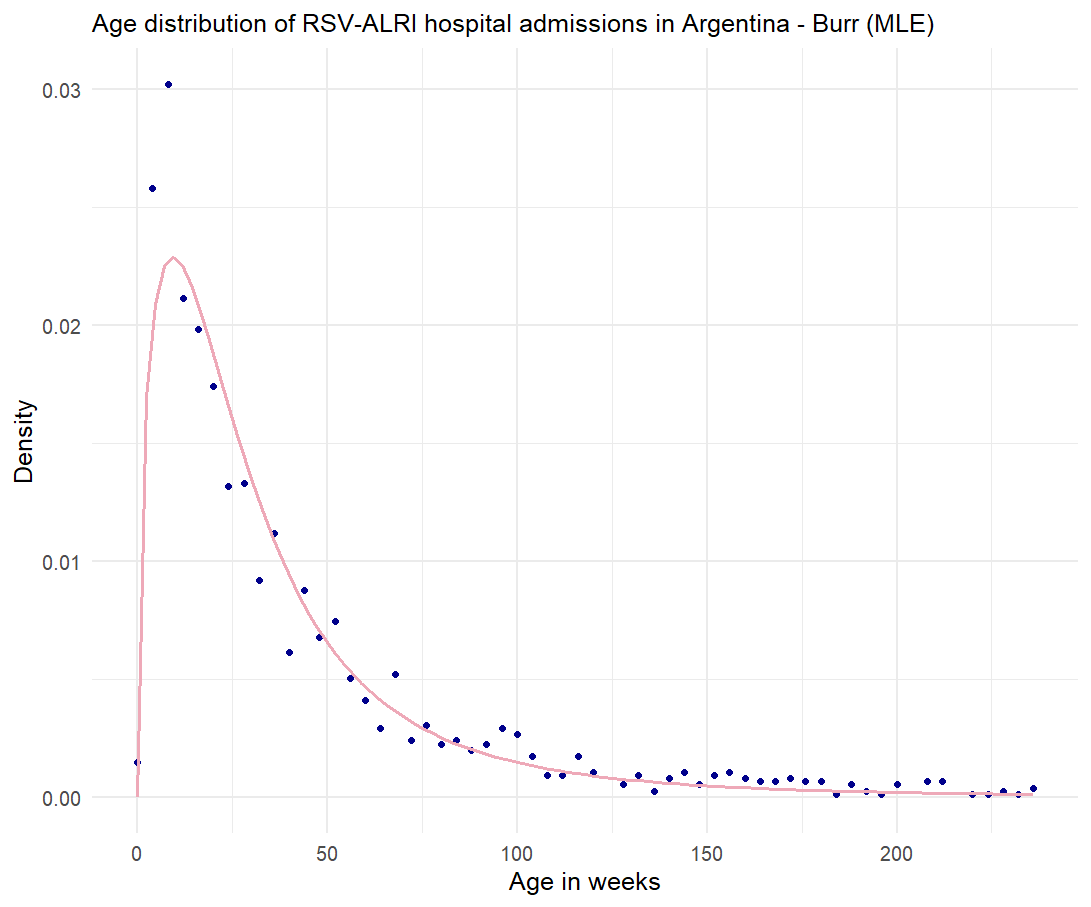


**Supplementary Figure 2. Clinical and budget impact of RSV prevention strategies compared to no intervention in Argentina. Period 2025-2034**

**Supplementary Table 1. Lifetime costs and effects of maternal vaccination ($25/dose, 69 % efficacy, 6 months protection) and infant mAb ($25/dose, 77 % efficacy, 5 months protection) in Argentina. Period 2025–2034.**

|  | **No RSV intervention** | **Infant monoclonal antibody (RSV mAb)** | **Maternal vaccine (maternal RSV vaccine)** |
| --- | --- | --- | --- |
| **Lifetime costs and effects** |  |  |  |
| Non-severe RSV cases <5yrs | 3,856,021 | 3,347,397 | 3,527,560 |
| Non-severe RSV clinic visits <5yrs | 2,774,225 | 2,408,294 | 2,537,912 |
| Severe RSV cases <5yrs | 521,459 | 409,482 | 418,331 |
| Severe RSV clinic visits <5yrs | 445,554 | 349,877 | 357,438 |
| Severe RSV hospital admissions <5yrs | 311,641 | 244,720 | 250,008 |
| Severe RSV deaths <5yrs | 4,628 | 3,178 | 3,315 |
| DALYs (discounted*) | 124,241 | 85,450 | 89,191 |
| RSV strategy costs (discounted*) | - | $ 134,420,041 | $ 124,660,739 |
| Government healthcare costs (discounted*) | $ 257,459,185 | $ 207,333,754 | $ 214,014,047 |
| Societal healthcare costs (discounted*) | $ 283,757,121 | $ 228,843,772 | $ 236,328,668 |
| **Differences (comparator = no vaccine)** | - |  |  |
| Non-severe RSV cases <5yrs | - | -508,624 | -328,461 |
| Non-severe RSV clinic visits <5yrs | - | -365,931 | -236,313 |
| Severe RSV cases <5yrs | - | -111,977 | -103,128 |
| Severe RSV clinic visits <5yrs | - | -95,678 | -88,116 |
| Severe RSV hospital admissions <5yrs | - | -66,921 | -61,633 |
| Severe RSV deaths <5yrs |  | -1,451 | -1,313 |
| Percent reduction in severe RSV deaths <5yrs | - | -31,30% | -28,37% |
| DALYs (discounted*) | - | -38,791 | -35,050 |
| RSV strategy costs (discounted*) | - | $ 134,420,041 | $ 124,660,739 |
| Government healthcare costs (discounted*) |  | -$ 50,125,431 | -$ 43,445,138 |
| Societal healthcare costs (discounted*) | - | -$ 54,913,349 | -$ 47,428,453 |
| **Cost (US$) per DALY averted (comparator = no vaccine)** |  |  |  |
| *Government cost perspective* |  |  |  |
| Cost (discounted*) |  | $ 84,294,610 | $ 81,215,601 |
| DALYs averted (discounted*) | - | 38,791 | 35,050 |
| Cost per DALY averted (discounted*) | - | $ 2,173 | $ 2,317 |
| *Societal cost perspective* |  |  |  |
| Cost (discounted*) | - | $ 79,506,692 | $ 77,232,286 |
| DALYs averted (discounted*) | - | 38,791 | 35,050 |
| Cost per DALY averted (discounted*) | - | $ 2,050 | $ 2,204 |

**This would be dominated (extended dominance) by RSV mAb because RSV mAb would achieve health benefit more efficiently. However, we could note the substantial overlapping uncertainty i.e. subtle changes in parameters (price, efficacy etc) could lead to a different rank order, hence presenting both compared to nothing.

**Supplementary Table 2. Lifetime costs and effects of maternal vaccination ($100/dose, 69 % efficacy, 6 months protection) and infant mAb ($100/dose, 77 % efficacy, 5 months protection) in Argentina. Period 2025–2034.**

|  | **No RSV intervention** | **Infant monoclonal antibody (RSV mAb)** | **Maternal vaccine (maternal RSV vaccine)** |
| --- | --- | --- | --- |
| **Lifetime costs and effects** |  |  |  |
| Non-severe RSV cases <5yrs | 3,856,021 | 3,347,397 | 3,527,560 |
| Non-severe RSV clinic visits <5yrs | 2,774,225 | 2,408,294 | 2,537,912 |
| Severe RSV cases <5yrs | 521,459 | 409,482 | 418,331 |
| Severe RSV clinic visits <5yrs | 445,554 | 349,877 | 357,438 |
| Severe RSV hospital admissions <5yrs | 311,641 | 244,720 | 250,008 |
| Severe RSV deaths <5yrs | 4,628 | 3,178 | 3,315 |
| DALYs (discounted*) | 124,241 | 85,450 | 89,191 |
| RSV strategy costs (discounted*) | - | $ 510,701,740 | $ 473,523,247 |
| Government healthcare costs (discounted*) | $ 257,459,185 | $ 207,333,754 | $ 214,014,047 |
| Societal healthcare costs (discounted*) | $ 283,757,121 | $ 228,843,772 | $ 236,328,668 |
| **Differences (comparator = no vaccine)** | - |  |  |
| Non-severe RSV cases <5yrs | - | -508,624 | -328,461 |
| Non-severe RSV clinic visits <5yrs | - | -365,931 | -236,313 |
| Severe RSV cases <5yrs | - | -111,977 | -103,128 |
| Severe RSV clinic visits <5yrs | - | -95,678 | -88,116 |
| Severe RSV hospital admissions <5yrs | - | -66,921 | -61,633 |
| Severe RSV deaths <5yrs |  | -1,451 | -1,313 |
| Percent reduction in severe RSV deaths <5yrs | - | -31,30% | -28,37% |
| DALYs (discounted*) | - | -38,791 | 35,050 |
| RSV strategy costs (discounted*) | - | $ 510,701,740 | $ 473,523,247 |
| Government healthcare costs (discounted*) |  | -$ 50,125,431 | -$ 43,445,138 |
| Societal healthcare costs (discounted*) | - | -$ 54,913,349 | -$ 47,428,453 |
| **Cost (US$) per DALY averted (comparator = no vaccine)** |  |  |  |
| *Government cost perspective* |  |  |  |
| Cost (discounted*) |  | $ 460,576,309 | $ 430,178,109 |
| DALYs averted (discounted*) | - | 38,791 | 35,050 |
| Cost per DALY averted (discounted*) | - | $ 11,873 | $ 12,273 |
| *Societal cost perspective* |  |  |  |
| Cost (discounted*) | - | $ 455,788,391 | $ 426,194,794 |
| DALYs averted (discounted*) | - | 38,791 | 35,050 |
| Cost per DALY averted (discounted*) | - | $ 11,750 | $ 12,160 |

*Future costs/effects were discounted at a rate of 3% per year. Cost was calculated as follows: vaccine program cost - healthcare costs averted

**This would be dominated (extended dominance) by RSV mAb because RSV mAb would achieve health benefit more efficiently. However, we could note the substantial overlapping uncertainty i.e. subtle changes in parameters (price, efficacy etc) could lead to a different rank order, hence presenting both compared to nothing.

**Supplementary Table 3. Lifetime costs and effects of maternal vaccination ($50/dose, 69 % efficacy, 6 months protection) and infant mAb ($50/dose, 77 % efficacy, 5 months protection) in Argentina. Single cohort (2025).**

|  | **No RSV intervention** | **Infant monoclonal antibody (RSV mAb)** | **Maternal vaccine (maternal RSV vaccine)** |
| --- | --- | --- | --- |
| **Lifetime costs and effects** |  |  |  |
| Non-severe RSV cases <5yrs | 387,006 | 335,958 | 354,151 |
| Non-severe RSV clinic visits <5yrs | 278,432 | 241,706 | 254,795 |
| Severe RSV cases <5yrs | 52,336 | 41,907 | 41,985 |
| Severe RSV clinic visits <5yrs | 44,718 | 35,115 | 35,874 |
| Severe RSV hospital admissions <5yrs | 31,278 | 24,561 | 25,092 |
| Severe RSV deaths <5yrs | 465 | 319 | 333 |
| DALYs (discounted*) | 14,159 | 9,739 | 10,165 |
| RSV strategy costs (discounted*) | - | $ 29,648,265 | $ 473,523,247 |
| Government healthcare costs (discounted*) | $ 29,404,209 | $ 23,679,423 | $ 24,445,161 |
| Societal healthcare costs (discounted*) | $ 32,407,675 | $ 26,136,065 | $ 26,994,134 |
| **Differences (comparator = no vaccine)** | - |  |  |
| Non-severe RSV cases <5yrs | - | -51,048 | -32,854 |
| Non-severe RSV clinic visits <5yrs | - | -36,726 | -23,637 |
| Severe RSV cases <5yrs | - | -12,362 | -10,350 |
| Severe RSV clinic visits <5yrs | - | -10,563 | -8,844 |
| Severe RSV hospital admissions <5yrs | - | -6,717 | -6,186 |
| Severe RSV deaths <5yrs |  | -146 | -132 |
| Percent reduction in severe RSV deaths <5yrs | - | -31,40% | -28,39% |
| DALYs (discounted*) | - | -4,420 | -3,994 |
| RSV strategy costs (discounted*) | - | $ 29,648,265 | $ 27,507,882 |
| Government healthcare costs (discounted*) |  | -$ 5,724,786 | -$ 4,959,372 |
| Societal healthcare costs (discounted*) | - | -$ 6,271,610 | -$ 5,413,917 |
| **Cost (US$) per DALY averted (comparator = no vaccine)** |  |  |  |
| *Government cost perspective* |  |  |  |
| Cost (discounted*) |  | $ 23,923,4791 | $ 22,548,510 |
| DALYs averted (discounted*) | - | 4,420 | 3,994 |
| Cost per DALY averted (discounted*) | - | $ 5,413 | $ 5,646 |
| *Societal cost perspective* |  |  |  |
| Cost (discounted*) | - | $ 23,376,655 | $ 22,093,965 |
| DALYs averted (discounted*) | - | 4,420 | 3,994 |
| Cost per DALY averted (discounted*) | - | $ 5,289 | $ 5,532 |

**Supplementary Table 4. Variation in disease events based on vaccine coverage scenarios.**

|  |  | **Vaccine coverage** | | | |
| --- | --- | --- | --- | --- | --- |
| **Disease events** | | **100%** | **90%** | **60%** | **50%** |
| **RSV non-severe visits** | RSV mAb | -84,216 (-3,50%) | -41,162 (-1,71%) | +94,536 (+3,93%) | +139,768 (+5,80%) |
|  | maternal RSV vaccine | -70,370 (-2,78%) | -38,777(-1,53%) | +56,000 (+2,21%) | +87,593 (+3,46%) |
| **RSV**  **hospitalization** | RSV mAb | -15,800 (-6,46%) | -7,528 (-3,08%) | +17,289 (+7,06%) | +25,561 (+10,44%) |
|  | maternal RSV vaccine | -20,764 (-8,31%) | -12,524 (-5,01%) | +12,195 (+4,88%) | +20,435 (+8,17%) |
| **RSV**  **deaths** | RSV mAb | -343 (-10,79%) | -163 (-5,13%) | +375 (+11,79%) | +554 (+17,43%) |
|  | maternal RSV vaccine | -442 (-13,33%) | -267 (-8,05%) | +260 (+7,84%) | +529 (+15,95%) |

**Absolute and percentage variation compared to base case values**

**Supplementary Table 5. Analysis of different vaccination strategies.**

| **Efficacy for fixed duration** | | | | | | |  |
| --- | --- | --- | --- | --- | --- | --- | --- |
| **Strategies** | **Reduction in admissions aged <5 yrs admissions** | | **Doses required per 1% reduction** | | **Increased dose efficiency compared to annual strategy** | |  |
|  |  |  |  |  |  |  |  |
|  |  |  |  |  |  |  |  |
|  | **Vax** | **mAb** | **Vax** | **mAb** | **Vax** | **mAb** |  |
| Jan only | 3% | 2% | 2299 | 3439 | 19% | -16% |  |
| Jan-Feb | 6% | 5% | 2094 | 2683 | 26% | 9% |  |
| Jan-Mar | 10% | 10% | 1816 | 2008 | 36% | 32% |  |
| Jan-Apr | 16% | 17% | 1581 | 1622 | 44% | 45% |  |
| Jan-May | 21% | 23% | 1466 | 1454 | 48% | 51% |  |
| **Jan-Jun** | **23%** | **26%** | **1577** | **1548** | **45%** | **48%** |  |
| Jan-Jul | 24% | 27% | 1793 | 1756 | 37% | 41% |  |
| Jan-Aug | 24% | 27% | 2041 | 1998 | 28% | 32% |  |
| Jan-Sep | 24% | 27% | 2296 | 2248 | 19% | 24% |  |
| Jan-Oct | 24% | 27% | 2548 | 2494 | 10% | 16% |  |
| Jan-Nov | 25% | 27% | 2739 | 2744 | 4% | 7% |  |
| Jan-Dec | 26% | 27% | 2845 | 2956 | - | - |  |

| **Efficacy wanes gradually** | | | | | | |  |
| --- | --- | --- | --- | --- | --- | --- | --- |
| **Strategies** | **Reduction in admissions aged <5 yrs admissions** | | **Doses required per 1% reduction** | | **Increased dose efficiency compared to annual strategy** | |  |
|  |  |  |  |  |  |  |  |
|  |  |  |  |  |  |  |  |
|  | **Vax** | **mAb** | **Vax** | **mAb** | **Vax** | **mAb** |  |
| Jan only | 2% | 3% | 3059 | 2657 | -21% | -15% |  |
| Jan-Feb | 4% | 5% | 2791 | 2455 | -10% | -6% |  |
| Jan-Mar | 8% | 10% | 2216 | 1986 | 13% | 14% |  |
| Jan-Apr | 14% | 17% | 1729 | 1581 | 32% | 32% |  |
| Jan-May | 21% | 25% | 1471 | 1365 | 42% | 41% |  |
| **Jan-Jun** | **24%** | **28%** | **1526** | **1422** | **40%** | **39%** |  |
| Jan-Jul | 25% | 30% | 1709 | 1593 | 33% | 31% |  |
| Jan-Aug | 26% | 30% | 1919 | 1783 | 24% | 23% |  |
| Jan-Sep | 26% | 31% | 2122 | 1963 | 16% | 15% |  |
| Jan-Oct | 27% | 32% | 2303 | 2123 | 9% | 8% |  |
| Jan-Nov | 28% | 33% | 2434 | 2233 | 4% | 4% |  |
| Jan-Dec | 29% | 35% | 2539 | 2319 | - | - |  |

**Seasonal approach. Efficacy waning**

In this model, that incorporated a gradual waning of protection, the strategy resulted in a 17% reduction in impact for maternal RSV vaccine (Supplementary Table 5 and Supplementary Figure 4C), accompanied by a 40% increase in dose efficiency compared with a year-round immunization program. Similarly, for the use of RSV mAb, the reduction in clinical impact would be 20%, with a 39% increase in dose efficiency (Supplementary Table 4 and Figure 4d).

**Supplementary Figure 4. Analysis clinical impact and cost-effectiveness. Reduction of admissions and doses for each 1% reduction. Efficacy wanes gradually**


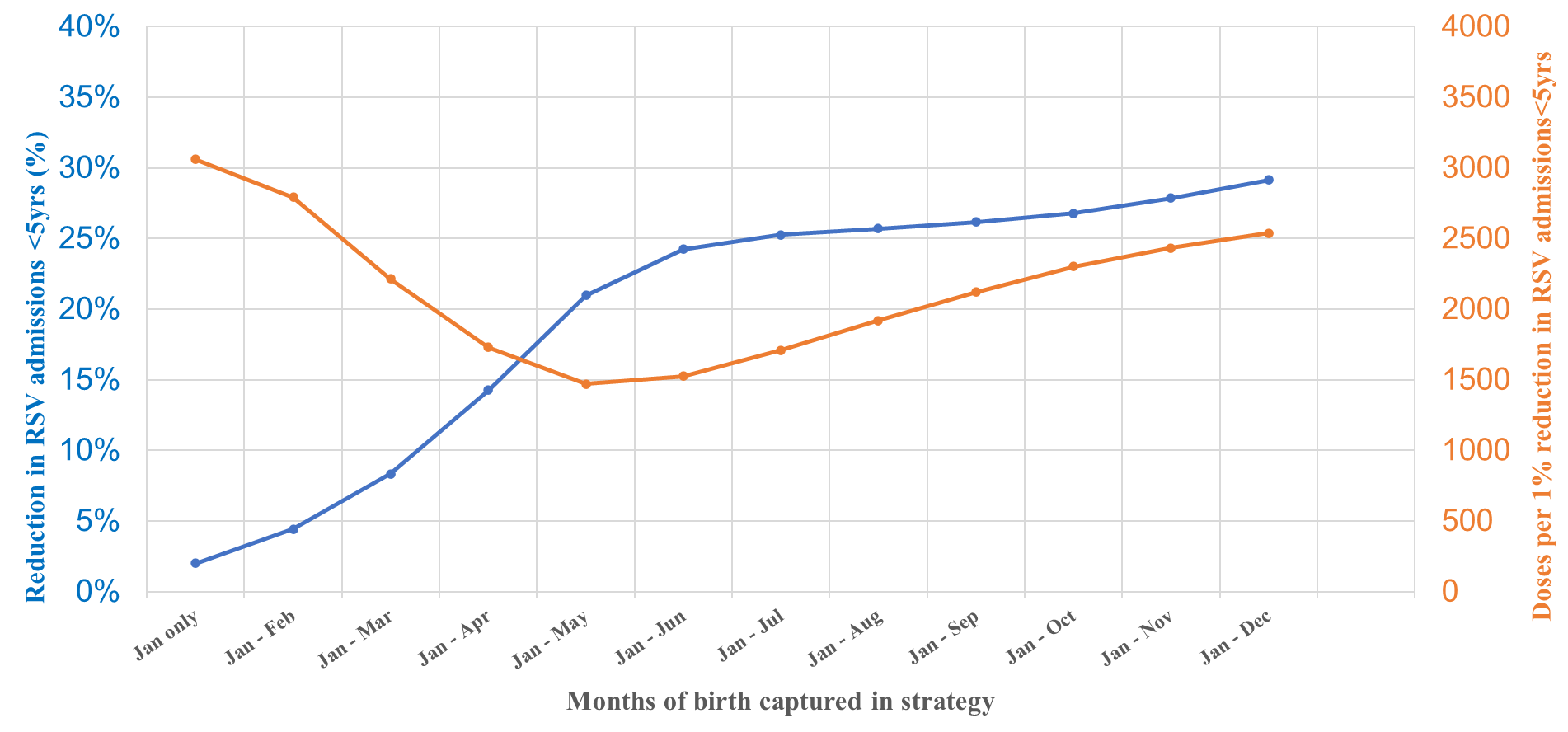


**C**

**D**


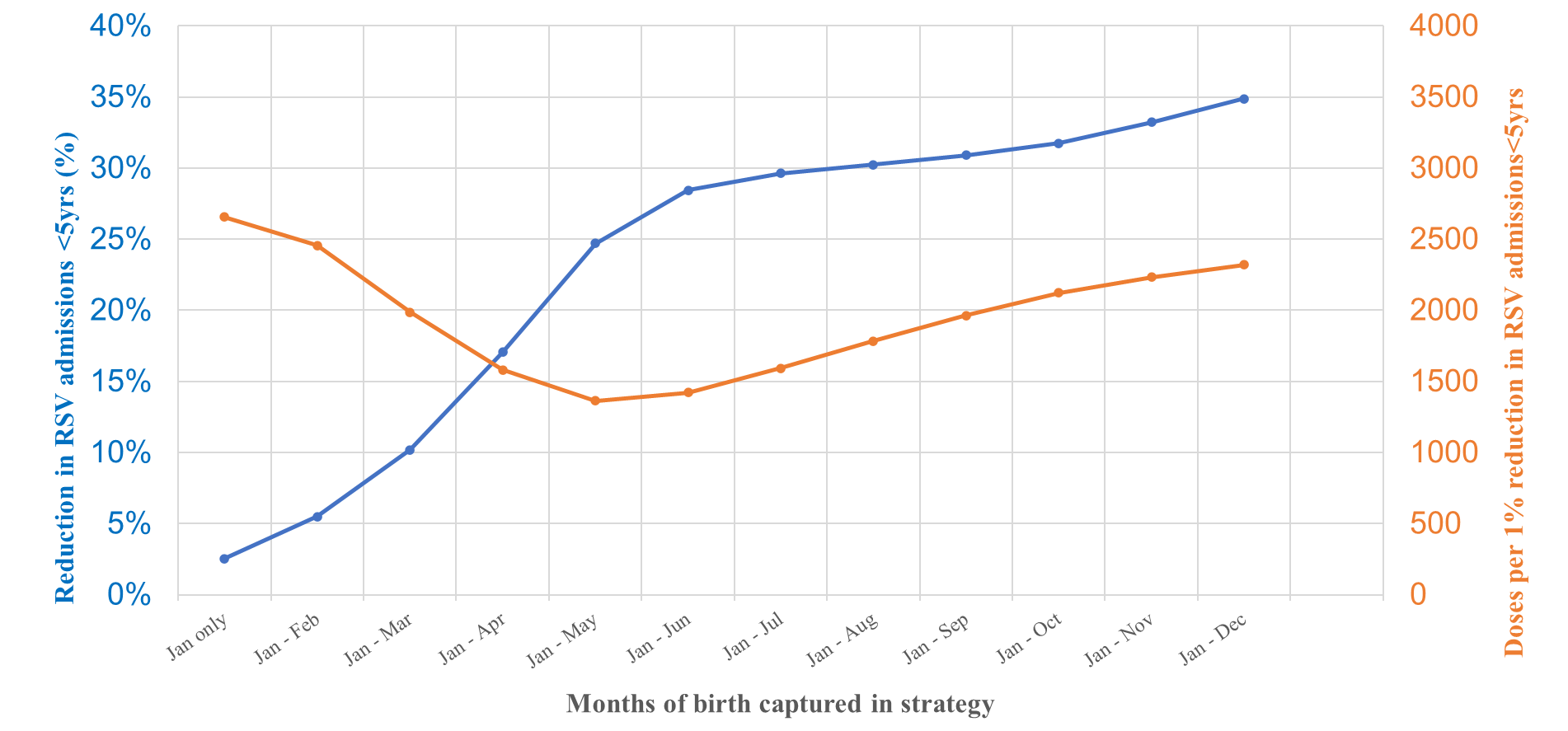


**[C] RSV mAb, [D] Maternal Vaccine**

**References**

1. Li Y, Wang X, Blau DM, et al. Global, regional, and national disease burden estimates of acute lower respiratory infections due to respiratory syncytial virus in children younger than 5 years in 2019: a systematic analysis. *Lancet*. 2022;399(10340):2047-2064. doi:10.1016/S0140-6736(22)00478-0
2. Caballero MT, Bianchi AM, Grigaites SD, et al. Community Mortality Due to Respiratory Syncytial Virus in Argentina: Population-based Surveillance Study. Clin Infect Dis. 2021;73(Suppl_3):S210-S217. doi:10.1093/cid/ciab497
3. Geoghegan S, Erviti A, Caballero MT, et al. Mortality due to Respiratory Syncytial Virus. Burden and Risk Factors. Am J Respir Crit Care Med. 2017;195(1):96-103. doi:10.1164/rccm.201603-0658OC
4. Dvorkin J, Sosa E, Vodicka E, et al. Cost of illness due to respiratory syncytial virus acute lower respiratory tract infection among infants hospitalized in Argentina. BMC Public Health. 2024;24(1):427. Published 2024 Feb 10. doi:10.1186/s12889-024-17878-3
